# Research highlights for issue 5: the role of the microbiome in shaping evolution

**DOI:** 10.1111/eva.12167

**Published:** 2014-05-27

**Authors:** Britt Koskella

Over the past century, the study of genetics has revolutionized our understanding of life on earth. Our knowledge of trait heritability from parent to offspring has been central to predict the trajectory of evolution, studying disease, and successful breeding of crops and animals. The field of genetics continues to grow in leaps and bounds due to next-generation sequencing, metagenomic approaches, genetic engineering, a better understanding of epigenetics, and, most recently, the creation of synthetic chromosomes (Annaluru et al. 2014). Despite these advances, however, there is still an active debate regarding how much variation in phenotype is explained by nature (the genome) versus nurture (the environment; recently reviewed in Lynch and Kemp 2014). In addition, it is increasingly apparent that a significant proportion of the so-called missing heritability may be explained by host-associated microbial communities, the microbiome.

The microbiome of eukaryotes has been associated with traits ranging from disease susceptibility to digestion to behavior and even holds the potential to drive speciation (Brucker and Bordenstein 2013). This rapidly growing field already has its own journal (‘Microbiome,’ established in 2013) and has been the focus of a recent special issue of Microbial Ecology on ‘Nature's microbiome’ (Russell et al. 2014), in which 28 research groups present new ideas and data on the composition and function of the microbiome and on how microbe–microbe and host–microbe interactions might shape evolution. Among the recent headlines, we have seen a role for soil-associated microbes in creating the taste of particular wine vintages (Bokulich et al. 2014) and good evidence that immune defense is modulated both directly and indirectly by our microbiota (recently reviewed in Abt and Pamer 2014). In addition, work by Maggie Wagner and colleagues on a wild relative of Arabidopsis has uncovered the key role of soil microbiota both in shaping flowering time and in influencing the intensity of selection on flowering time (Wagner et al. 2014).

Given the complexity of studying the human microbiome, much of our current understanding comes from work carried out on germ-free mice. A recent study by Jeremiah Faith and coauthors, which introduced 94 bacterial consortia of diverse sizes chosen at random from human fecal samples, was able to uncover key roles of the microbiota in inflammatory responses, obesity and variation in metabolites in mouse hosts (Faith et al. 2014). Determining how human microbiomes are shaped and how they may have coevolved with the population requires very large datasets to account for the great variation in diet, geography, race, and lifestyle among individuals. However, recent insight into how microbiota may have changed due to urbanization has come from Stephanie Schnorr and colleagues, who sequenced the microbiome of individuals from the Hadza hunter–gatherer community in Tanzania (Schnorr et al. 2014). They find evidence suggesting that, relative to individuals from urban communities in Italy, the Hadza microbiota is typically more species rich and lacks the typically common *Bifidobacterium*. Of course, as recently discussed by Eva Boon and collaborators, the importance of variation in microbiota composition among individuals and populations is less about what species are there than it is about what genes are there (Boon et al. 2014). This is both because of redundancy in function among microbial species and also due to the ability of bacteria to horizontally transmit genetic material among genomes, such that one population can readily evolve a new function simply by acquiring the necessary genes.

Given the current open questions regarding the function and composition of human microbiota, we are not yet at the stage of developing artificial communities as treatment for disease. However, in extreme cases of *Clostridium difficile* infection, doctors have been turning to fecal transplants as a way of resetting the microbiome of their patients with remarkably high success rates. Susana Fuentes and colleagues recently tracked changes in the microbiome of patients before, during and after such transplants and found a marked and long-lasting increase in microbial diversity after the transplant (Fuentes et al. 2014). It remains to be determined whether success rate is affected by interactions between the host genotype and the transplant microbiota, but we can look to data from other organisms for such clues. For example, Marie-Lara Bouffaud and coauthors report a significant relationship between rhizobacterial communities and genetic distance of their plant hosts, and this relationship held when looking only at single bacterial species (Bouffaud et al. 2014). On the other hand, work by Julie Reveillaud and colleagues found no clear signature of host relatedness in explaining the microbiota associated with coral hosts, although their data do suggest species-specific microbiota communities even across geographically distant deep sea populations (Reveillaud et al. 2014).

Another potential application of microbiome research is the use of ‘prebiotics,’ particular dietary fibers, to manipulate the composition of the microbiota. Amandine Everard and coauthors tested the impact of prebiotic treatment on mice that were fed high-fat diets and found that the differing composition in microbiota of treated mice acted to counteract inflammation and metabolic disorders induced by the high-fat diet (Everard et al. 2014). However, to fully translate the burgeoning microbiome data into practical applications, such as the use of pre- or probiotics to prevent/treat disease or to alter organismal phenotype in a predictable way, we need to untangle the complexity of social interactions among microbes more generally. This idea has been highlighted by Helen Leggett and coauthors, who review the wide range of ways in which microbes interact within their eukaryotic hosts (Leggett et al. 2014). The review emphasizes that better insight into microbe–microbe social evolution, both within and between species, will be central to better predicting the evolution of virulence, drug resistance, and the spread of infectious disease. The idea of harnessing information about social interactions, including those between microbes, to design novel treatments has been coined ‘Hamiltonian medicine’ and recently conceptualized by Bernard Crespi and colleagues (Crespi et al. 2014).

Together, the wealth of new data emphasizes that microbiota play central roles in shaping the health, ecology, and evolution of their hosts. The application of microbiota research is currently hindered by the complexity of the interactions (both among microbes and between the microbiota and the host), but the potential for application of this knowledge seems limitless.
